# Screening a Suspected Malingerer Using the Symptom Checklist-90-Revised and Visual Analogue Pain Scale Questionnaires

**DOI:** 10.7759/cureus.62904

**Published:** 2024-06-22

**Authors:** Kinan Sawar, Lana Sawar, Vaishnavi Reddy, Abdullah Sahyouni, Amar Sawar

**Affiliations:** 1 Internal Medicine, Wayne State University School of Medicine, Detroit, USA

**Keywords:** healthcare cost reduction, symptom checklist-90-revised, diagnostic screening tool, mva (motor vehicle accident), visual analogue scale, malingering

## Abstract

Malingering in healthcare leads to a significant financial burden, so identifying patients who may be more likely to malinger is a critical step in minimizing the ever-growing cost of healthcare in the United States. Malingering is a clinical diagnosis with no well-established diagnostic tests. General guiding principles exist to determine whether or not a patient is malingering, but there is no well-established set of guidelines that can be used in common to identify malingering. Our team cared for a 51-year-old black, female patient who presented to an outpatient clinic due to generalized pain following a motor vehicle accident (MVA). The patient's symptomatology, clinical progression, and imaging results were discordant with one another, which prompted clinical suspicion of malingering. After careful deliberation, the care team suspected that the patient was malingering. Therefore, the clinical management was limited to a conservative pain management regimen and minimal clinical follow-up to avoid unnecessary healthcare expenditures. This article aims to discuss general principles and specific strategies for how a clinician can approach a case of suspected malingering.

## Introduction

The definition of a malingering patient feigns physical or psychological symptoms to gain external benefits, monetary benefits, access to particular medications, or to avoid personal responsibilities (time off work, time off school, etc.) [1]. The ability to identify malingering patients is crucial to ensure high-value, high-quality care is provided to patients, as the estimated financial burden of malingering is roughly $20 billion per year [2]. Clinicians can often miss the diagnosis of malingering as it requires a clinician to view the patient as someone capable of malintent. Albeit rare, sometimes this line of thinking must be taken if the clinical scenario one is presented with leads to likely suspicion of patient malintent. Particular clinical scenarios prompt the inclusion of this diagnosis in a differential diagnosis list. For example, approximately one-third of personal injury cases and 8% of medical cases were deemed probable malingering cases based on a large sample of clinical reports [3]. The diagnosis of malingering does not have a well-established diagnostic approach or algorithm. Therefore, a literature review was performed to evaluate and synthesize clinicians' approaches to diagnosing malingering. Considering the nature of this clinical case, only articles discussing malingering in the context of patients experiencing motor vehicle accidents (MVAs) were considered.

A search of PubMed using the terms "malingering" AND "motor vehicle accident" yielded 6 results. This search was expanded using a PubMed search for relevant MeSH terms. The MeSH term "Accidents, Traffic" [Mesh] was added to the search such that the final search query was "malingering" AND ("motor vehicle accident" OR "Accidents, Traffic" [Mesh]), which resulted in 37 publications. The abstracts were manually evaluated for studies that included diagnostic strategies for detecting malingering in patients who experienced MVAs. Of the 37 results, 2 relevant papers were identified, including 1 case study and 1 randomized clinical trial.

Parker et al. described a case study of a female patient involved in an MVA [4]. The patient had a fractured pelvis, which was medically managed, and once the patient was deemed to be capable of rehabilitation, she was told to begin slowly increasing physical activity, but she refused. This patient had an exaggerated gait that changed significantly from day to day, random bouts of confusion that were not consistent with one another, and entirely normal physical exam and imaging findings, which probed physicians to diagnose her as malingering. This case study exemplified a classic scenario in which a malingering patient can present in a way that is contradictory to a patient's verbalized complaints.

The most impactful study in our search was a randomized controlled trial by Wallis et al., which compared pain scores between college students who were asked to simulate chronic pain 6 months after an MVA to actual whiplash injury patients [5]. This publication is classified as SORT level 2 evidence [6]. The primary purpose of the study was to determine if the Symptom Checklist-90-Revised (SCL-90-R) questionnaire is an effective screening tool in differentiating patients who are malingering from those who have actually experienced whiplash injuries. The study enrolled 40 pain-free college students and 132 patients with whiplash injuries. The college students were selected from the visual arts department as it was assumed that these students would likely be unfamiliar with the diagnostic or compensation criteria for whiplash injuries. These students were told to answer all questionnaires in a way they believed would best simulate a patient who experienced a whiplash injury. Each group was given 3 questionnaires: the Symptom Checklist-90-Revised (SCL-90-R), the Visual Analogue Scale (VAS), and the McGill Pain Questionnaire. The students simulating whiplash injury scored statistically significantly higher on all the subscales of both the SCL-90-R and the VAS than patients with true whiplash injuries. In contrast, the two groups' pain scores on the McGill Pain Questionnaire were not statistically significant. This article is the first of its kind to assess the use of these questionnaires to screen for malingering.

Although the SCL-90-R is the only questionnaire loosely used to screen for malingering behavior, the researchers decided to give both groups these other two questionnaires to determine if there is any statistically significant correlation between the scores on these other two popular pain scales and malingering behavior. The SCL-90-R is a 90-item questionnaire describing the attributes of various physical and psychological symptoms the patient may be experiencing [7]. The questionnaire is divided into subscales that describe pain-related measures. The SCL-90-R was never previously assessed before this study as a screening tool for specifically malingering. However, it has been used in the past to assess for psychological distress in patients with chronic pain who were involved in a whiplash injury. The students simulating whiplash injury scored statistically significantly higher on the SCL-90-R and the VAS subscales. Patients with legitimate whiplash injury experienced increased scores on the somatization, depression, and anxiety subscales of the SCL-90-R relative to other subscales. The pain scores on the McGill Pain Questionnaire were not statistically significant between the two groups.

Unfortunately, this study did not discuss any quantitative cutoff values that could be used to screen patients as possible malingerers in a clinical setting. Nevertheless, because people faking whiplash injuries are, on average, more likely to overreport pain across all SCL-90-R subscales than someone with an actual injury and more likely to report higher scores on the VAS, this information can be used to help identify malingering patients.

## Case presentation

A 51-year-old black female with a past medical history of diabetes, hyperlipidemia (HLD), hypertension (HTN), obesity, and chronic low back pain presented to the clinic with a complaint of worsening cervical neck pain, low back pain, muscle spasms, headache, and generalized muscle soreness eight days after an MVA. This patient first visited the emergency department (ED) the same day of the accident. At that point, it was assessed that she had acute cervical strain and multiple acute contusions of undocumented location. At the ED, the patient had CT head, CT cervical spine, lumbosacral spine x-ray, abdominal x-ray, chest x-ray, and right shoulder x-ray performed. All imaging had negative or insignificant findings. 

Following the ED, she came into the clinic four days after the accident. At that time, it was assessed that the patient was still recovering from the muscle contusions and needed a more effective pain management regimen based on the physical exam and ROS. The patient's prescription drug monitoring program record was checked for previous history of controlled substance use, and no record of previous drug use was uncovered. The patient's medical history did not mention any previous history of back pain, neck pain, or muscle spasms. Given this information, the patient was prescribed meloxicam 7.5 mg up to three times daily, cyclobenzaprine hydrochloride 10 mg every 8 hours as needed, and a ketorolac shot 30 mg in the clinic. The patient was also advised to apply hot and cold compresses on her sore muscles, starting with ice for the next two days, followed by heat for the following two days. 

The patient came back to the clinic four days later, still complaining of cervical and lumbar back pain, back spasms, and generalized muscle soreness. She said she had been taking meloxicam three times per day, but this was "not helping at all" with the pain. At this time, the patient was informed she could take acetaminophen 650 mg up to four times daily as needed. She had not used acetaminophen in the last four days because she said "it did nothing." Interestingly, the patient stated she was unable to recall whether the 30 mg ketorolac shot four days prior helped alleviate the pain. She said that she tried using the hot and cold compresses but that they only helped as a distraction. The patient did mention that cyclobenzaprine hydrochloride was effective in controlling her back spasms. The patient was frustrated that "nothing [was] helping [her] pain and that it had been getting worse." The physical exam was insignificant. There were no neurological or musculoskeletal findings other than generalized paravertebral tenderness. 

At this second office visit, the patient sought out three months of time off of work, which raised suspicion as to the motive of her clinical visits. After a discussion with the patient, she filled out the Family and Medical Leave Act (FMLA) paperwork, allowing her time off work for two weeks. She works as an office clerk and was cleared to work from home. She was given a referral for physical therapy and was started on a 10-day course of hydrocodone 5 mg twice daily. Lastly, she was told to call our office if her symptoms worsen. This patient's insignificant physical exam and imaging suggested that she may be malingering, but there was no definitive method to make this determination at the time of the patient's first and second office visits. Retrospective assessment of the patient's clinical course led us to conclude that this patient was likely malingering. Ideally, more office visits would be necessary to definitively determine if the patient is malingering. However, given the patient's rapid pursuit of an external reward, such as time off work in this case, the treating physician needed to make a quick decision on whether to continue treating the patient as if she were experiencing significant pain.

## Discussion

Further questioning of the patient's MVA was necessary to assess for malingering for the patient in question. This questioning should have included information such as where she was bruised, where exactly she was hit in the head, whether she went to the ER immediately, whether she had applied for disability before, and whether she had driven a car since the accident as she claimed to be traumatized by cars. Her answers should be coherent with one another. A thorough chart review can help look for a pattern of malingering-like behavior if she had previously visited other providers before presenting to you. In this case, she had no previous history of this type of behavior documented. Upon physical exam, the clinician can distract the patient while palpating her paravertebral muscles to determine if she really is experiencing pain and prevent her from having the opportunity to simulate the experience of pain. This approach aligns with methodologies to detect malingering in existing studies. For example, Greer et al. emphasize the significance of collecting detailed patient histories and cross-checking information to screen for malingering [8]. Moreover, a literature review of malingering emphasizes using the physical examination to assess pain authenticity. This includes observing weakness not seen in other activities and disablement disproportionate to objective physical exam, laboratory, and imaging findings [9].

**Figure 1:**
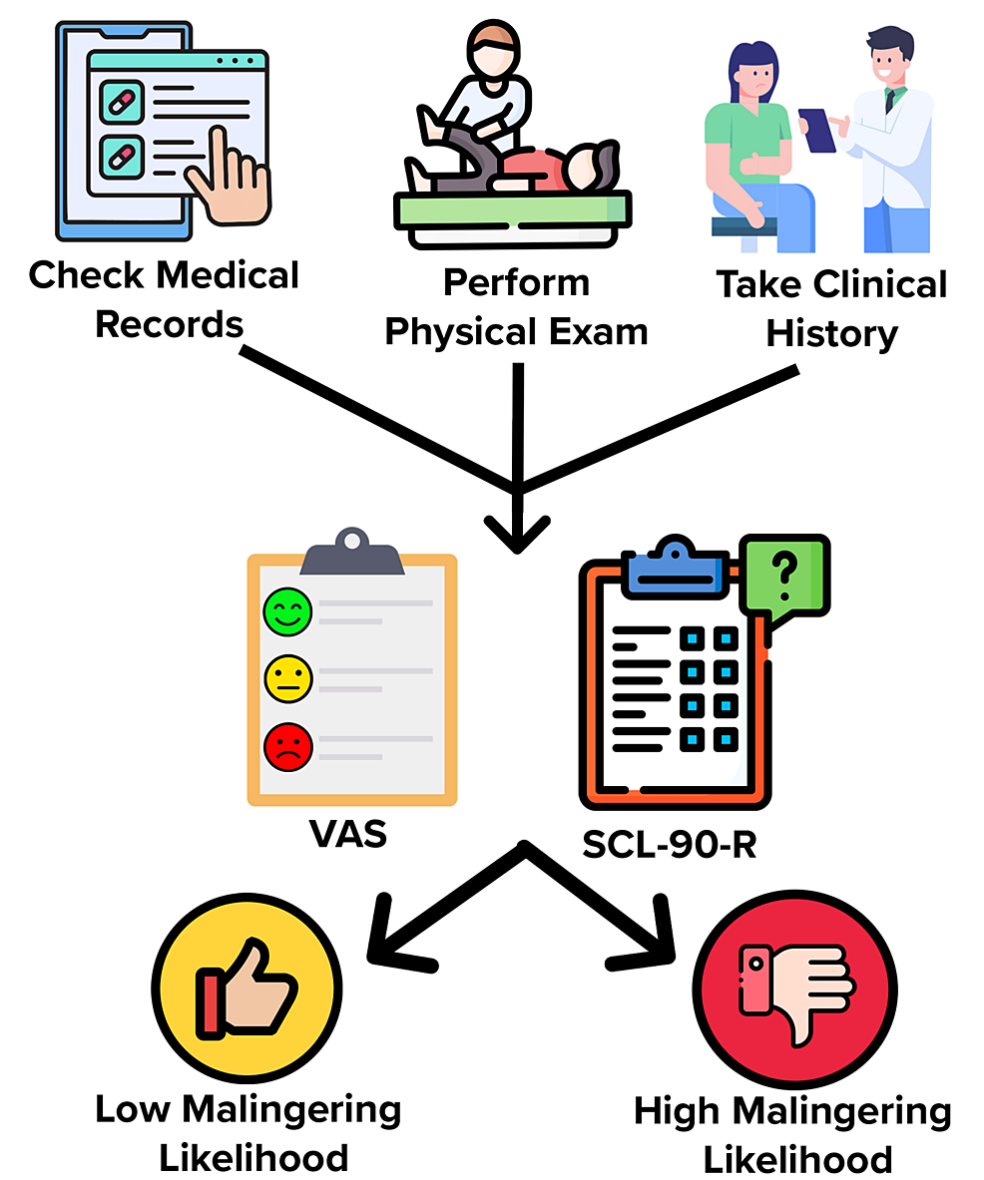
Diagnostic Pathway for Evaluating Motor Vehicle Accident (MVA) Patients Suspected of Malingering Any MVA patient that is suspected of malingering should have their medical records, physical exam, and clinical history cross-checked in order to determine whether there is discordance between the findings of these information sources. If suspicion grows after this cross-review, have the patient complete the Visual Analogue Scale (VAS) and the Symptom Checklist-90-Revised (SCL-90-R) questionnaires to determine whether the patient has a low or high likelihood of malingering. Remember, patients simulating whiplash injury score significantly higher on all the subscales of the SCL-90-R and the VAS than patients with true whiplash injuries. Image Credits: Kinan Sawar

Tests other than the SCL-90-R and VAS should be used in cases of suspected cognitive malingering. Other studies have shown that the use of the coin-in-hand test, Tests of Memory Malingering (TOMM), Rey Fifteen-Item Test (FIT), and Word Memory Test (WMT) are effective in evaluating patients suspected of cognitive malingering [10-13]. Specifically, in MVA cases, the patient's healthcare team can use the SCL-90-R and VAS to help determine if the patient is feigning or exaggerating pain if the scores are discordant with the patient's clinical presentation. If the patient is malingering, then the patient will have high scores on both these questionnaires and high scores in all subcategories of the SCL-90-R. Evaluation using these questionnaires helps a physician stratify patients into a high or low likelihood of malingering but cannot be used in isolation to make this assessment (Figure 1). In this case, after the patient got her FMLA paperwork approved following the first visit, the patient's demeanor changed, which was evident during the second visit. If a patient were to rate his or her pain an 8/10 and receive a tramadol injection to relieve the pain, that patient would certainly be aware of whether this treatment reduced the pain. Our care team asked her just four days later, so she likely would not have forgotten. Malingerers generally stop complaining after achieving the external benefit they seek, FMLA in this case. A unique aspect of this clinical scenario is that the patient had the entirety of her treatment covered by her car insurance because of Michigan's no-fault insurance policy, which provides coverage for health expenses related to MVAs, providing even more incentive for MVA patients in Michigan to malinger. Considering all possibilities of external reward given the circumstances of a patient's case is important to rule in or rule out patient malingering. 

## Conclusions

It can be difficult to accurately assess whether a patient is malingering post-MVA or not. The best way to determine whether a patient is malingering in this case is to use a combination of quantitative and qualitative metrics. For example, a patient experiencing maximal pain cannot sit completely still. Next, a physician can use a series of physical exam techniques to gather more information on whether this patient is likely malingering or not. This includes repeating the physical exam multiple times and documenting inconsistencies between exams. Next, the physician should ask the patient to describe the pain (location, quality, severity) and transcribe this information to track it over time to determine if the patient's description stays consistent throughout the patient experiencing any symptoms related to the MVA. Questioning the patient about symptoms not related to the MVA might lead the patient to falsely confirm that he or she is experiencing these arbitrary symptoms to convince the provider that a medical issue exists. After performing the tasks as described, it is up to the physician to make an executive decision as to whether or not the patient is feigning physical or psychological symptoms for external gain after integrating the results of the SCL-90-R, VAS, and qualitative assessment of the patient's unique clinical situation.
